# Effects of low-frequency repetitive transcranial magnetic stimulation combined with cerebellar continuous theta burst stimulation on spasticity and limb dyskinesia in patients with stroke

**DOI:** 10.1186/s12883-021-02406-2

**Published:** 2021-09-24

**Authors:** Dawei Li, Aixia Cheng, Zhiyou Zhang, Yuqian Sun, Yingchun Liu

**Affiliations:** Department of Neurological Rehabilitation, Shengli Oilfield Central Hospital, No. 31, Jinan Road, Dongying, 257000 Shandong China

**Keywords:** Repetitive transcranial magnetic stimulation, Cerebellar continuous theta burst stimulation, Spasticity, Dyskinesia, Stroke

## Abstract

**Background:**

Repetitive transcranial magnetic stimulation (rTMS) has been reported to treat muscle spasticity in post-stroke patients. The purpose of this study was to explore whether combined low-frequency rTMS (LF-rTMS) and cerebellar continuous theta burst stimulation (cTBS) could provide better relief than different modalities alone for muscle spasticity and limb dyskinesia in stroke patients.

**Methods:**

This study recruited ninety stroke patients with hemiplegia, who were divided into LF-rTMS+cTBS group (n=30), LF-rTMS group (n=30) and cTBS group (three pulse bursts at 50 Hz, n=30). The LF-rTMS group received 1 Hz rTMS stimulation of the motor cortical (M1) region on the unaffected side of the brain, the cTBS group received cTBS stimulation to the cerebellar region, and the LF-rTMS+cTBS group received 2 stimuli as described above. Each group received 4 weeks of stimulation followed by rehabilitation. Muscle spasticity, motor function of limb and activity of daily living (ADL) were evaluated by modified Ashworth Scale (MAS), Fugl-Meyer Assessment (FMA) and Modified Barthel Index (MBI) scores, respectively.

**Results:**

The MAS score was markedly decreased, FMA and MBI scores were markedly increased in the three groups after therapy than before therapy. In addition, after therapy, LF-rTMS+cTBS group showed lower MAS score, higher FMA and MBI scores than the LF-rTMS group and cTBS group.

**Conclusion:**

Muscle spasticity and limb dyskinesia of the three groups are all significantly improved after therapy. Combined LF-rTMS and cTBS treatment is more effective in improving muscle spasticity and limb dyskinesia of patients after stroke than LF-rTMS and cTBS treatment alone.

## Introduction

Stroke is the onset of neurological deficits caused by acute focal injury to the central nervous system (CNS) due to vascular causes, and is a leading cause of worldwide disability and death [1]. Varying degrees of limb dyskinesia are the most common consequences of stroke [2]. Spasticity, usually defined as a velocity-dependent elevation of muscle tone because of amplified stretch reflexes, is manifested in 65% of survivors who suffer a stroke [3]. It limits the mobility of patients and may worsen the long-term disability. Current treatments for post-stroke spasticity and limb dyskinesia include physiotherapy, pharmacotherapy, surgical procedures, etc. [4, 5]. However, each of these approaches has limitations. Therefore, the development of new interventions is urgently needed.

Transcranial magnetic stimulation (TMS) is a feasible, painless, noninvasive, and well tolerated brain stimulation technique that has been widely used in clinical diagnosis, prognosis, research, and treatment [6, 7]. Repetitive TMS (rTMS) is a train of TMS pulses that continuously act on the local brain with constant stimulus intensity. Conventional rTMS patterns include low-frequency rTMS (LF-rTMS, < 1 Hz) and high-frequency rTMS (HF-rTMS, >1 Hz) [8]. Contralesional LF-rTMS can inhibite, and ipsilesional HF-rTMS can promote the local cortical excitability [9, 10]. The theta burst stimulation (TBS) is a novel rTMS consisting of three pulse bursts at 50 Hz. it is divided into two forms, continuous TBS (cTBS) and intermittent TBS (iTBS), which play inhibitory and facilitatory roles in local cortical excitability, respectively [11]. Thus, both of LF-rTMS and cTBS stimulate the motor cortical areas (M1 areas) on the unaffected side to achieve inhibitory effects on excitation, which in turn balances the affected side.

In addition to the differences in TMS stimulation patterns, the different sites of TMS stimulation can also affect the therapeutic efficacy. At present, the stimulation sites for TMS application and research mainly focus on the cerebral cortex. In addition, cerebellar cTBS is a recently discovered new treatment modality that does not act directly on the cerebral cortex but on the cerebellum. It is known that cerebellum is involved in motor control and has role in movement disorders [12, 13]. TMS stimulation of the cerebellum modulates not only cerebellar cortical function, but also the primary M1 area and other relevant functional areas in the remote brain [14, 15]. Therefore, the cerebellum has gradually emerged as a new stimulus target for higher-order functional regulation of the brain. Therefore, this study also focused on the pattern of stimulation of the cerebellum by cTBS.

It has been found that LF-rTMS stimulation of the unaffected cerebral hemisphere improves muscle spasticity and dyskinesia of the upper limb and lower extremity after stroke [16, 17]. In addition, previous studies have shown that cerebellar cTBS modulates motor cortex excitability [14] and ameliorates the motor symptoms of several motor disorders such as levodopa-induced dyskinesias [18], cervical dystonia [19] and essential tremor [20]. However, the role of cerebellar cTBS and combined cerebellar LF-rTMS and cTBS in muscle spasticity and limb dyskinesia after stroke remains unknown.

Thus, this study aimed to analyze and compare the effects of combined LF-rTMS and cerebellar cTBS and different modalities alone in relieving muscle spasticity and limb dyskinesia in post-stroke patients. This study will provide a more effective technique for rehabilitation of muscle spasticity and limb dyskinesia in post-stroke patients.

## Materials and methods

### Participants and groups

This study was approved by the Ethics Committee of Shengli Oilfield Central Hospital. In addition, informed consent was obtained from patients or family members, and signed informed consent was obtained. The participants were 90 hemiplegic patients with stroke (cerebral infarction and/or intracerebral hemorrhage), who admitted to Shengli Oilfield Central Hospital from January 2020 to December 2020. Inclusion criteria for patients were as follows: 1) patients were diagnosed according to the updated definition of stroke for the 21st century [1] by a neurologist, and were confirmed to be ischemic or hemorrhagic stroke of the unilateral cerebral hemisphere by cranial CT or MRI imaging examination; 2) the lesion was in the middle cerebral artery territory.; 3) the myofunctional research center (MRC) grades of muscle strength in the affected limb were ≥ 3, and elbow and knee flexion and extension movements can be completed; 4) the patients were first onset, with disease duration between 1 and 6 months and age between 40 and 70 years; 5) patients had no obvious cognitive dysfunction and could cooperate with treatment, examination, and efficacy assessment; 6) patients had no severe proprioception, vestibular or visual dysfunction; 7) patients were mildly to moderately impaired in balance (20 ≤ Berg balance function score ≤ 40); 8) the muscle tone in the paralyzed limb was > 0 grade as assessed by modified Ashworth Scale (MAS). Patients were excluded if they 1) had lacunar or silent cerebral infarcts; 2) had a worsening condition and could not continue to cooperate with treatment; 3) were severely paralyzed and had impairments in cognitive function or verbal communication; 4) had severe cardiovascular, hepatic, renal and other diseases; 5) had osteoarthropathy and pain affecting the patient's normal activities; 6) had contraindications to rTMS treatment (such as intracranial metal implants, pacemakers, skull defects, or history of seizures); 7) had a history of drug use (such as sedation, antidepressants) that altered cortical excitability; 8) were pregnant women.

All patients were randomly divided into treatment (LF-rTMS+cTBS group) and control groups, and the control group was divided into a control group 1 (LF-rTMS group) and a control group 2 (cTBS group), with 30 patients in the three group. The present study was an open study. Meanwhile, patients in each group were well coordinated to receive rehabilitation therapy. Besides, the patients were all right-handed.

### Test and stimulation procedures before treatment

Resting motor threshold (RMT) was determined for each patient before treatment. Patients adopt the sitting position and wear positioning caps on their heads. We then placed a “Circular” treatment coil of rTMS in the primary motor cortex (M1 Region - the site where stimulation with a single TMS pulse elicits maximal macroscopic movement of the first interosseous dorsal muscle of the contralateral hand) of the affected side of brain. Manual control of the single pulse output was used to observe whether the patient had a response in the contralateral finger or jaw. RMT was defined as the minimum stimulus intensity that elicited a motor evoked potential (MEP) > 50 UV over the first dorsal interosseous muscle on the affected side (detected with an electrode needle) in at least 5 of 10 consecutive stimulations. If maximal magnetic stimulation still fails to evoke a motor response, RMT can be determined by selecting stimulation points (mirror areas) at the same site on the unaffected side. The patient was asked to lie in the prone position and was relaxed as much as possible, and the patients remained awake at all times during the course of the treatment. A stimulation coil was placed in the brain M1 region, tangential to the scalp. The coil is then secured with a brace and the patient is instructed not to move his head so as not to stimulate site change.

### TMS treatment in the control and treatment groups

In the LF-rTMS group, 1 Hz rTMS was applied to the contralesional M1 region of the brain at a stimulation intensity of 80% of RMT, a stimulation time of 5 s, and five stimuli with an interval of 1 s. The number of repetitions was 200, the total number of stimuli was 1000, and the total treatment time was 20 min.

In the cTBS group, 50 Hz cTBS was applied to the right cerebellar hemisphere, and the stimulation point was located 3 cm lateral to the midline and 1 cm below the inion. The cTBS was applied with 80% of active motor threshold (AMT) of stimulation intensity, 20 s of stimulation time, 100 stimuli, 3 pulses within clusters, and no time interval between stimuli. This was repeated 4 times with a total number of stimuli of 1200 and a total treatment time of 80 s.

In the LF-rTMS+cTBS group, patients received the same stimulation treatment first as the patients in the LF-rTMS group and then the same stimulation treatment as the patients in the cTBS group, and the total time of treatment required 21 min and 20 s. Patients in these 3 groups were treated 1 time per day, 6 days per week (1 day off), for a total of 4 weeks. During the application of rTMS, doctors carefully monitored all patients.

### Rehabilitation therapy

After the TMS stimulation, the patients received the rehabilitation therapy, which included rehabilitative training and acupuncture therapy. In rehabilitative training, physiotherapists primarily train movements of the lower limbs and trunk; an occupational therapists primarily train patients in upper extremity motor function and fine finger function and an occupational therapy involves training such as pushing sand, screw wringing, nine hole post, tying buttons. Therapists increased training intensity and difficulty as appropriate based on patient recovery. Rehabilitation training was performed for 4 weeks, 5 days a week, 2 times a day for 40 min. In addition, an acupuncture therapy was performed for 4 weeks, 5 days a week, once a day for 20 min.

### Muscle tone test

The MAS, one of the main methods to assess muscle spasticity clinically, is a graded rating scale [21]. Spasticity was evaluated before and 4 weeks after treatment in both groups. We selected the elbow and wrist joints of the upper extremities, the knee and ankle joints of the lower extremities as observation subjects. In addition, we converted 1 + from the original rank score to 1.5 to facilitate statistical analysis of the metrology score. On the MMAS score, 0 indicates no increase in muscle tone and 4 indicates a partial flexion extension stiffness in the affected segment.

### Motor function evaluation

The Fugl-Meyer Assessment (FMA) scale was used to assess motor function of limbs, including upper extremity function, hand function, and lower extremity function. Motor function was evaluated before treatment, 4 weeks after treatment in both groups. Designed by Fugl-Meyer et al. in 1975 [22], FMA is a global assessment measure used to quantitatively assess limb recovery in hemiplegia after stroke. The FMA has a total of 50 items, each rated on a 3-point scale (0 = unable to complete a certain action, 1= able to partially complete a certain action, and 2 = able to adequately complete a certain action).

### Assessment of activity of daily living (ADL)

The ADL of stroke patients before and 4 weeks after treatment was assessed with the Modified Barthel Index (MBI), and the aim was to evaluate the independent self-care ability and walking ability of patients [23]. It includes a total of 10 items, and the total score of 10 items is 100 points. A higher score means a stronger ADL.

### Statistical analysis

SPSS 22.0 (IBM Corp.) and GraphPad Prism 7.0 software (GraphPad Software, Inc.) were used to perform the statistical analyses. Comparison between categorical variables was conducted by Chi-square test. The metrology data were expressed as mean ± standard deviation (SD). Differences between two groups of metrology data were compared using Student’s t-test and between multiple groups of metrology data were compared using one-way ANOVA followed by Tukey’s post hoc test. Each experiment was repeated at least 3 times. *P* < 0.05 was defined statistically significant.

## Results

### Baseline characteristics of the patients

The clinical characteristics of the patients were summarized in Table 1. There were no significant differences among the three groups in general data, including age, gender, time since the stoke, stroke type, paretic side, or BBS scores (all *P* > 0.05). In addition, no significant differences were found in MAS score, FMA score, and MBI score among patients who received TMS before treatment (all *P* > 0.05). Thus, the three groups of patients were comparable.

**Table 1:** Baseline characteristics of the patients with stroke

Variables	LF-rTMS (n=30)	cTBS (n=30)	LF-rTMS+cTBS (n=30)	*P* value
Age (years)	57.60±7.40	55.13±7.90	56.77±8.58	0.479
Gender (males, n)	19	18	20	0.866
Time since the stoke (months)	3.80±1.71	3.67±1.84	3.63±1.85	0.931
Stroke type				0.935
Hemorrhage	7	6	6	
Infarction	23	24	24	
Paretic side				0.861
Right	11	9	10	
Left	19	21	20	
BBS score	30.53±6.06	29.40±6.44	29.03±5.97	0.618
MAS score	6.83±1.70	6.93±1.79	6.80±1.97	0.958
FMA score	46.10±17.81	45.57±16.55	45.10±17.49	0.975
MBI score	46.87±25.30	49.47±23.80	48.63±22.11	0.911

*LF-rTMS* low-frequency repetitive transcranial magnetic stimulation, *cTBS* continuous theta burst stimulation, *BBS* Berg Balance Scale, *MAS* Modified Ashworth Scale, *FMA* Fugl-Meyer Assessment, *MBI* Modified Barthel Index

### Effects of LF-rTMS and cTBS on muscle tone

**Fig. 1:**
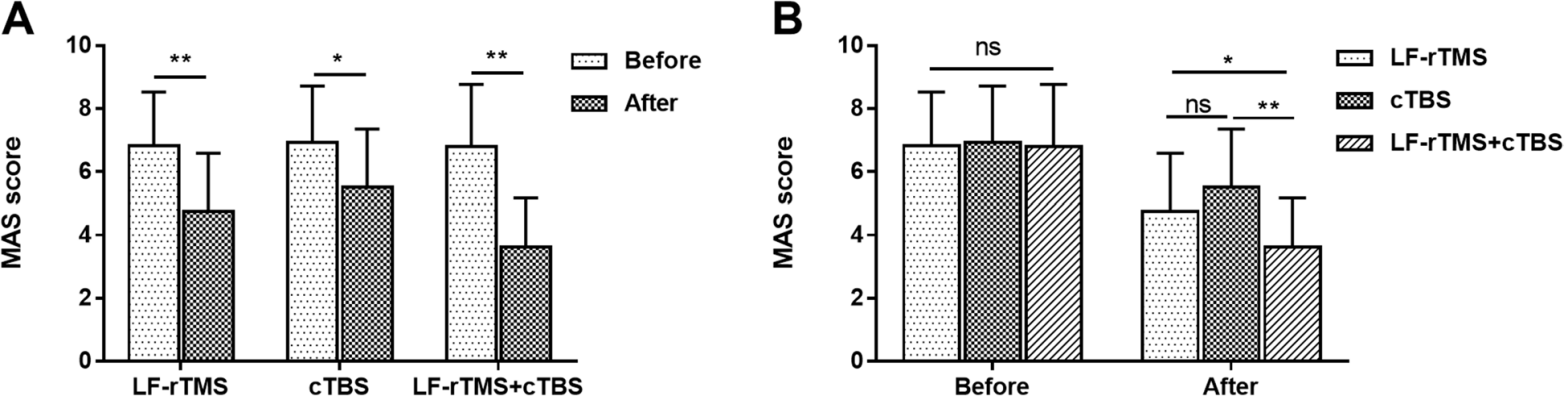
Effects of LF-rTMS and cTBS on muscle tone. A. MAS score was significantly decreased after treatment than before treatment in LF-rTMS group, cTBS group and LF-rTMS+cTBS group. **B**. LF-rTMS+cTBS group had markedly lower MAS score than LF-rTMS group and cTBS group after treatment. *P < 0.05, **P < 0.01 vs. before treatment or LF-rTMS group or cTBS group; ns, no significant. LF-rTMS, low-frequency repetitive transcranial magnetic stimulation; cTBS, continuous theta burst stimulation; MAS, Modified Ashworth Scale

MAS score was used to assess muscle tone. The results in Fig. 1A showed that in all group, the MAS score was significantly lower after treatment than before treatment (all *P* < 0.05). As shown in Fig. 1B, there was no significant MAS score difference among the three groups before treatment. After treatment, no significant difference was found in MAS score between the LF-rTMS group and the cTBS group, and the MAS score of LF-rTMS combined with cTBS group was markedly lower than that of LF-rTMS group and cTBS group (all *P* < 0.05).

### Effects of LF-rTMS and cTBS on motor function

**Fig. 2:**
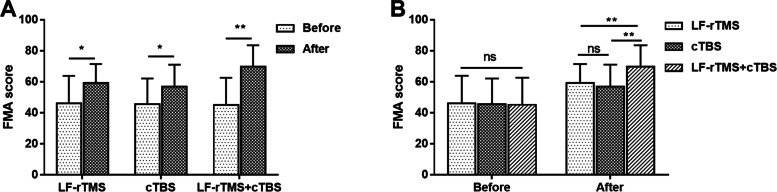
Effects of LF-rTMS and cTBS on motor function. **A**. FMA score was higher after treatment than before treatment in LF-rTMS, cTBS and LF-rTMS+cTBS groups. **B**. After treatment, LF-rTMS+cTBS group had markedly higher FMA score than LF-rTMS and cTBS groups. *P < 0.05, **P < 0.01 vs. before treatment or LF-rTMS group or cTBS group; ns, no significant. LF-rTMS, low-frequency repetitive transcranial magnetic stimulation; cTBS, continuous theta burst stimulation; FMA, Fugl-Meyer Assessment

FMA score was used to evaluate the motor function. The three groups had markedly increased FMA score after treatment compared with before treatment (Fig. 2A, all *P* < 0.05). As can be seen from Fig. 2B, no significant difference was observed in FMA score among three groups before treatment. After treatment, no significant difference was observed in FMA score between LF-rTMS group and cTBS group, and FMA score was markedly higher in patients receiving combined LF-rTMS and cTBS than that in those receiving single LF-rTMS or cTBS treatment (all *P* < 0.01).

### Effects of LF-rTMS and cTBS on ADL

**Fig. 3:**
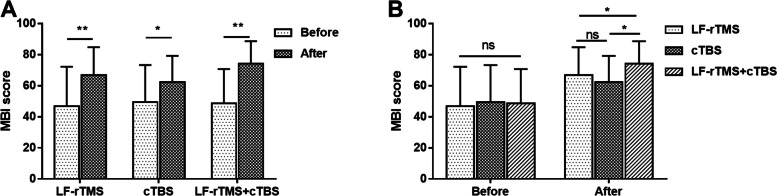
Effects of LF-rTMS and cTBS on ADL. **A**. Patients after treatment had markedly higher MBI score than those before treatment. **B**. MBI score was significantly higher in LF-rTMS+cTBS group than that in LF-rTMS and cTBS groups after treatment. *P < 0.05, **P < 0.01 vs. before treatment or LF-rTMS group or cTBS group; ns, no significant. LF-rTMS, low-frequency repetitive transcranial magnetic stimulation; cTBS, continuous theta burst stimulation; MBI, Modified Barthel Index; ADL, activity of daily living

The ADL was evaluated by MBI score. The results in Fig. 3A showed that patients receiving treatment had markedly higher MBI score than their pre-treatment score (all *P* < 0.05). As presented in Fig. 3B, no significant MBI score difference was found among the three groups before treatment. In addition, there was no significant difference between the LF-rTMS group and the cTBS group in terms of MBI score after treatment, however, MBI score was significantly higher in patients receiving combined treatment of LF-rTMS and cTBS than that in the LF-rTMS group and cTBS group after treatment (all *P* < 0.05).

## Discussion

This study demonstrated that both modes of LF-rTMS acting on the contralesional cerebral M1 and cTBS acting on the cerebellum improved muscle spasticity and limb dyskinesia in patients after stroke. In addition, the combined use of LF-rTMS and cerebellar cTBS produced better effects than either modality alone in improving muscle spasticity and dyskinesia of the limbs in patients after stroke.

Following rTMS stimulation of the cerebral cortex, cerebral nerves produce plasticity changes that facilitate recovery from stroke induced local brain injury. The results of Wan et al.’s meta-analysis showed that both contralesional low and ipsilesional high frequencies can exert therapeutic effects on limb dysfunction after stroke, but contralesional low frequencies are more effective than ipsilesional high frequencies [24], and this result is similar to the findings of shin et al. [25]. This study also utilized LF-rTMS to stimulate the contralesional side. In addition, an increasing number of studies on the effects of LF-rTMS on limb dysfunction after stroke have been reported [17, 26, 27]. Moreover, there have been studies on LF-rTMS for the treatment of limb spasticity in stroke, but not much. For example, 1 Hz rTMS on the unaffected hemisphere associated with physiotherapy can decrease the post-stroke upper limb spasticity [16]. The study by kakuda et al. similarly applies 1-Hz stimulation of the contralesional M1 region with a 15 day treatment, and demonstrates that 1 Hz rTMS combined with occupational therapy improve motor dysfunction induced by upper limb spasticity in stroke patients [28]. LF-rTMS (1 Hz) over the M1 of unaffected lower limbs can reduce lower limb spasticity and improve motor dysfunction caused by lower limb spasticity in stroke patients [17]. This study found that LF-rTMS stimulation of the contralesional side decreased MAS score and increased FMA and MBI scores in stroke patients after therapy, suggesting that LF-rTMS ameliorated patients’ muscle spasticity and limb dyskinesia. Thus, LF-rTMS stimulation of the contralesional side combined with rehabilitation therapy can improve muscle spasticity and limb dyskinesia in stroke patients.

The cerebellum is known to play crucial role in the motor control. Additionally, Cerebellar cTBS can modulate the corticospinal excitability through cerebellar-dentato-thalamo-cortical pathways [14, 15, 29], suggesting that cerebellar cTBS has the potential to affects spasticity. However, it does not necessarily follow that cerebellar stimulation affects spasticity, partly because the slight to moderate changes in MEP amplitude, which is often used as a marker for corticospinal excitability, is usually not enough to bring about spasticity or improvement of it. Thus, the effect of cerebellar cTBS on spasticity need to be further studied. In this study, we used MAS score to evaluate the spasticity. In addition, the ameliorative effects of cerebellar cTBS on motor symptoms in levodopa-induced dyskinesia [18, 30] and essential tremor [20] have been reported. Notably, some studies have shown that cerebellar cTBS improves motor symptoms in patients with dystonia [19, 31]. This study found the markedly reduced MAS score and markedly increased FMA and MBI scores in stroke patients after cerebellar cTBS treatment, suggesting the improvement of cerebellar cTBS on the muscle spasticity and limb dyskinesia, motor function of limb and ADL of patients after stroke. In addition, the cerebellar cTBS is known to be mainly applied in neuropsychiatric disorders and/or neurological domains such as multiple system atrophy [32], cognitive disorders [33], and memory consolidation in eyeblink classical conditioning [34]. Therefore, cerebellar cTBS has beneficial effects on muscle spasticity and limb dyskinesia in post-stroke patients.

We investigated whether combining LF-rTMS and cerebellar cTBS provides better relief of muscle spasticity and limb dyskinesia in post-stroke patients than LF-rTMS and cerebellar cTBS alone. The findings of the present study demonstrated that the combination of LF-rTMS and cerebellar cTBS ameliorated the muscle spasticity, motor function of limb and ADL of post-stroke patients with muscle spasticity and limb dyskinesia, and was more effective than LF-rTMS and cerebellar cTBS alone. Thus, combined LF-rTMS and cerebellar cTBS produced better improvements on the muscle spasticity and limb dyskinesia of stroke patients than the single LF-rTMS and cerebellar cTBS. Currently, some previous studies have shown that LF-rTMS can be combined with other treatments to improve spastic movement disorders in stroke patients [16, 28]. Notably, to our knowledge, this study is the first to investigate the effects of LF-rTMS combined with cerebellar cTBS on muscle spasticity and limb dyskinesia in patients after stroke.

The present study has certain limitations. First, the sample size was relatively small, and a larger sample size is needed in future studies. Second, we did not examine the long-term effects of treatment after completion of the study, and the persistence of these effects should also be investigated in future studies.

In conclusion, LF-rTMS combined with cerebellar cTBS show better efficacy than LF-rTMS/cerebellar cTBS in treating muscle spasticity and limb dyskinesia in patients with stroke. The combination of LF-rTMS and cerebellar cTBS may be a beneficial adjunctive technique for motor neurorehabilitation in stroke patients with muscle spasticity and limb dyskinesia.

## Data Availability

The data used to support the findings of this study are available from the corresponding author upon reasonable request.

## Abbreviations

TMS: Transcranial magnetic stimulation
rTMS: Repetitive transcranial magnetic stimulation
LF-rTMS: Low-frequency rTMS
cTBS: Continuous theta burst stimulation
ADL: Activity of daily living
FMA: Fugl-Meyer Assessment
MBI: Modified Barthel Index
RMT: Resting motor threshold
MEP: Motor evoked potential
